# Consumption with Large Sip Sizes Increases Food Intake and Leads to Underestimation of the Amount Consumed

**DOI:** 10.1371/journal.pone.0053288

**Published:** 2013-01-23

**Authors:** Dieuwerke P. Bolhuis, Catriona M. M. Lakemond, Rene A. de Wijk, Pieternel A. Luning, Cees de Graaf

**Affiliations:** 1 Product Design and Quality Management Group, Wageningen University, Wageningen, The Netherlands; 2 Food and Biobased Research, Consumer Science & Intelligent Systems, Wageningen University and Research, Wageningen, The Netherlands; 3 Division of Human Nutrition, Wageningen University, Wageningen, The Netherlands; Pennington Biomedical Research Center, United States of America

## Abstract

**Background:**

A number of studies have shown that bite and sip sizes influence the amount of food intake. Consuming with small sips instead of large sips means relatively more sips for the same amount of food to be consumed; people may believe that intake is higher which leads to faster satiation. This effect may be disturbed when people are distracted.

**Objective:**

The objective of the study is to assess the effects of sip size in a focused state and a distracted state on ad libitum intake and on the estimated amount consumed.

**Design:**

In this 3×2 cross-over design, 53 healthy subjects consumed ad libitum soup with small sips (5 g, 60 g/min), large sips (15 g, 60 g/min), and free sips (where sip size was determined by subjects themselves), in both a distracted and focused state. Sips were administered via a pump. There were no visual cues toward consumption. Subjects then estimated how much they had consumed by filling soup in soup bowls.

**Results:**

Intake in the small-sip condition was ∼30% lower than in both the large-sip and free-sip conditions (P<0.001). In addition, subjects underestimated how much they had consumed in the large-sip and free-sip conditions (P<0.03). Distraction led to a general increase in food intake (P = 0.003), independent of sip size. Distraction did not influence sip size or estimations.

**Conclusions:**

Consumption with large sips led to higher food intake, as expected. Large sips, that were either fixed or chosen by subjects themselves led to underestimations of the amount consumed. This may be a risk factor for over-consumption. Reducing sip or bite sizes may successfully lower food intake, even in a distracted state.

## Introduction

Obesity is an increasing problem in Western society. Overweight and obesity are the result of a long-term positive energy balance in which energy intake is higher than energy expenditure. There is growing evidence that oral processing is important in the regulation of food intake [1]–[10]. Foods that are consumed quickly and require minimal oral processing, such as beverages and foods low in fiber content, lead to higher ad libitum intake [5], [8], [9], [11], and therefore promote over-consumption.

Eating rate (g/min) is influenced by bite size [5], [12]. A number of studies have demonstrated a positive relationship between bite and sip size and the amount of food intake [4], [6], [7], [13]–[18]. Controlled experimental studies that used fixed bite/sip sizes showed a reduction in intake for smaller bites/sips. Bites of 5 g compared to bites of 15 g have led to a reduction of 30% in a chocolate dairy product [4] and in tomato soup [7]. Sips of 5 g compared to 20 g have led to a reduction of 29% and 16% in regular energy orangeade and no energy containing orangeade, respectively [6].

The bite or sip size and are determined by food properties [5], [19], [20], and by individual characteristics [19], [21]. Consuming small bites rather than large bites involves more bites for consumption of the same amount of food. Due to a relatively higher number of bites which is associated with more effort, small bites may lead lower food intake. Food intake has been shown to be highly influenced by external factors as visual cues, serving and portion size, and effort [13], [15], [22]–[24]. Effort can be considered as an external cue and may unconsiously infleunce food intake.

It is also possible that taking relatively more smaller bites affect peoples' assumption that intake is higher compared to fewer larger bites, and therefore lead to lower food intake. Beliefs about the amount consumed play an important role in satiation [25]–[28]. For example, information about calorie content [29]–[31], or manipulating the time of the day [32], influenced the amount of food intake. These findings stress the importance of cognitive factors on satiation.

Cognitive aspects of food intake may be disrupted when people are distracted during food consumption. Cognitive restraint eating behavior (i.e., chronic tendency to limit food intake to control body weight), was offset by distraction; food intake increased when listening to a detective story [33]. A number of studies have shown that distraction through activities such as watching television or eating with friends usually led to higher food intake [34]–[39]. It is possible that distraction during consumption is associated with impaired monitoring of the amount consumed by visual cues [37], [40]. Other regulators of food intake, such as number of bites, bite size, eating rate, or meal duration may also be affected by distraction. In a distracted state, people may unconsciously increase their number of bites which leads to higher food intake. Consumption with smaller bites in a distracted state, may therefore, be less effective in reducing food intake.

The objective of this study is to assess effects of sip size in both focused and distracted states on ad libitum intake. Subjects estimated the amount consumed after intake to determine if sip size affects the perceived food intake. We hypothesize that consumption with larger sips results in higher intake and underestimation of the amount consumed. We then hypothesize that the effect of sip size on food intake is diminished in a distracted state, and that subjects, generally, underestimate the amount consumed when they are distracted.

## Subjects and Methods

### Subjects

Fifty-seven subjects were recruited for participation, 53 of whom (33 males, 20 females) completed the study. Three subjects dropped out before the start of the study and one subject missed four sessions. Subjects were healthy, had normal weight (BMI 18.5 to 25 kg/m^2^, mean ± SD: 22±2 kg/m^2^), were aged between 18 and 35 y (mean ± SD: 22±3 y) and liked creamy tomato soup (pleasantness score >5 on a 9-point hedonic scale). Exclusion criteria were: restrained eating behavior (Dutch Eating Behavior Questionnaire (DEBQ) score men: >2.89, women: >3.39); an energy-restricted diet during the last two months; gained or lost >5 kg weight during the last year; lack of appetite; smoking; gastrointestinal illness; diabetes; thyroid disease, or any other endocrine disorder; or being pregnant or breast feeding. Subjects were informed that the research aimed to investigate the effect of distraction on flavor perception of soup. This study was conducted according to the guidelines laid down in the Declaration of Helsinki and all procedures involving human subjects were approved by the Medical Ethical Committee of Wageningen University. All subjects signed an informed-consent form before participation. This study was registered (NTR: 3091) with the Dutch trial registration at: www.trialregister.nl/trialreg/admin/rctview.asp?TC=3091.

### Experimental design

**Figure 1:**
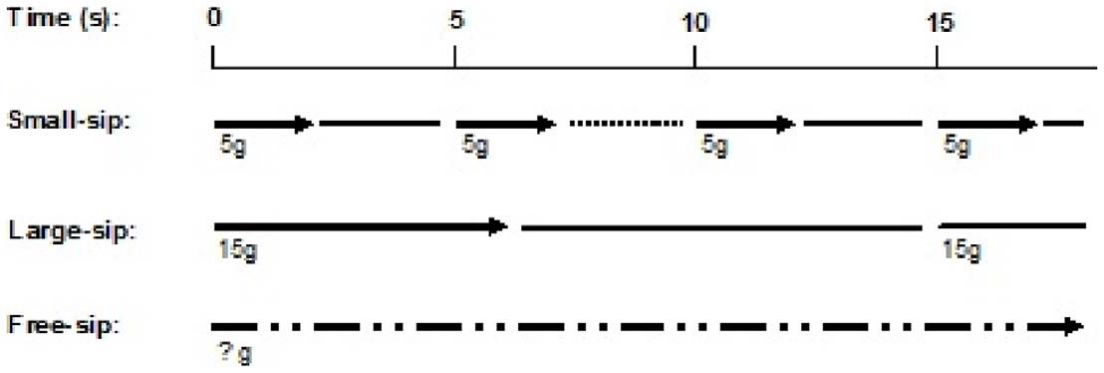
Sips and intervals in the three sip size conditions. 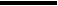
 = administration of soup, 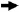 = instruction to swallow, 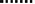 = pauses between sips, 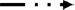 = regulation of sips and pauses by subjects themselves. All three conditions were presented in a focused and distracted state, resulting in six conditions. In the small-sip condition, sips of 5 g were exposed in 2 s (from the start of soup administration until swallowing) in pulses of 5 s. In the large-sip condition, sips of 15 g were exposed in 6 s in pulses of 15 s. In the free-sip condition, subjects were free to start and stop the pump, thereby determining sip sizes and frequency by themselves. In the small-sip and large-sip conditions, subjects heard an auditory signal when they received the soup and a double auditory signal when they had to swallow.

The experimental design is summarized in **Figure 1**. The study consisted of a 3×2 cross-over design. Subjects came to the lab seven times, including a first practice session. There were six different ad libitum intake conditions: small-sip, large-sip, and free-sip, presented in both a focused and a distracted state. The sip frequency was three times higher in the small-sip condition than in the large-sip condition, to keep the eating rate (g/min) similar. The eating rate was set at 60 g/min for both the small-sip and large-sip conditions. The oral residence duration (i.e., duration of food in the oral cavity) was 40 s/100 g for both small-sip and large-sip conditions. Subjects regulated the administration of the soup by themselves in the free-sip condition. They could start and stop the pump to determine sip sizes and frequencies.

In the ad libitum intake conditions, subjects first consumed a preload so that they would be less hungry prior to soup consumption [41]. It is possible that feelings of hunger would overrule sensory factors to terminate consumption.

### Control of sip sizes, intervals and swallowing

Subjects consumed soup through a food-grade tube (Saint-Gobain, Norprene, A-60-F, Charny, France) connected to a peristaltic pump (Watson-Marlow, type 323 Du, Watson-Marlow Bredel, Wilmington, MA, USA) to control sips and intervals. The tube ended in a pan of soup that was placed on a balance (Kern, type 440-49A, KERN & Sohn GmbH, Balingen, Germany) to record the amount consumed.

Subjects heard an auditory signal to inform them that the pump started working and they would receive soup in their mouths. They heard a double auditory signal when they had to swallow. The instruction to swallow was given 0.5 s after termination of sip administration. Subjects were instructed that it was very important to swallow at the double auditory signal before the start of each session.

The small-sip condition consisted of 5 s intervals. The large-sip condition consisted of 15 s intervals (Figure 1). Subjects received 15 g during the first 5.5 s of each interval and swallowed after 6 s. Subjects received 5 g during the first 1.5 s of each interval and swallowed after 2 s. In the free-sip condition, subjects could start and stop the pump by themselves. The pump rate was set at 2.5 g/s. This meant that, for example, a 4 s administration resulted in a 10 g sip. Subjects in the free-sip condition were instructed to swallow as soon as they stopped administration.

### Test foods

Tomato soup was used for this study. One kg of soup was made from 333 g sieved tomatoes (Heinz, Elst, The Netherlands), 662.7 g water, and 4.7 g salt (NaCl). The mixture was heated to 60°C. The calculated nutrient composition from the ingredients was: 0.57 g protein, 1.6 g carbohydrates, 0.03 g fat, 253 mg sodium and 38 kJ (9.1 kcal) energy per 100 g soup.

Raisin buns (local bakery) were used as preload. The nutrient composition was: 8 g protein, 52 g carbohydrates, 3 g fat, 300 mg sodium and 1120 kJ (268 kcal) energy per 100 g, according to the Dutch Food Composition Database (NEVO, version 2009/1.0). Each raisin bun weighed 22 g (246 kJ). The number of raisin buns was calculated at half of the energy provided by an average lunch in the Netherlands [42], equal to 11% energy of the daily energy need. The daily energy need for each subject was estimated by the Schofield I equation [43], taking into account: gender, age, weight and a physical activity level of 1.6. Sixteen subjects received 4 buns, 25 subjects received 5 buns, 12 subjects received 6 buns. Subjects were instructed to eat all the raisin buns they were served.

## Procedure

### First session

Subjects were familiarized with the experimental procedures during their first visit. They were seated in sensory booths. They received instructions and questions via a computer screen. Subjects received 45 g soup in both the small-sip and large-sip conditions, in randomized order. Subjects rated several sensory aspects after consumption of soup in both conditions to determine if sip size influences sensory characteristics.

### Sensory characteristics

The sensory characteristics rated in the first session were overall flavor intensity, saltiness, thickness, after-taste intensity, and “expected satiation”. All aspects were rated on a 100 mm visual analogue scale (VAS). The question that referred to overall flavor intensity, saltiness intensity and after-taste intensity was: “How strong is the flavor/saltiness/after-taste of this soup?” from “very weak” at the left end to “very strong” at the right end. The question that referred to thickness was: “How thick is the texture of this soup?” from “very thin” at the left end to “very thick” at the right end. The question that referred to “expected satiation” was: “How filling is this soup?” from “hardly filling” to “very much filling”.

### Ad libitum intake sessions

There were six lunch sessions for ad libitum intake of soup, with one week between sessions. The six conditions were presented at random to subjects. Subjects started by consuming the preload of raisin buns. Subjects were instructed to consume all raisin buns and were allowed to drink water. Subjects then paused for 20 minutes. During that time, subjects were allowed to study or read, but were not allowed to leave the sensory room.

After the pause, subjects received instructions and questions via a computer screen. Before ad libitum intake, subjects first rated appetite and hedonic aspects, as described below. Subjects could push a button on the computer screen to start soup consumption. The pan and balance were placed on the experimenters' side of the sensory booth, so there were no visual cues of the amount consumed. Subjects were instructed to terminate consumption any time when they felt they had enough. The mean (± SD) initial temperature of the soup was 55±3°C and the mean end temperature was 48±3°C.

Subjects were instructed to stay in the sensory booths for at least 15 minutes in both the focused and the distracted states. A visual warning signal popped up on the laptop screen to inform subjects that the 15 minutes had passed. This prevented subjects from leaving the research area other for than being satiated with the soup.

### Focus versus distraction

Subjects in the focused state were instructed to focus on the taste and flavor of the soup. Subjects in the distracted state were told they would see a short (∼15 min) animation film (“Pat and Mat”, *in Dutch*: “Buurman en Buurman”) during consumption and would answer questions about the film afterwards. This was done to ensure they focused on the film. There were six different films randomized between conditions and subjects. Subjects answered between 8 to 11 questions about the film. The film was started once subjects started consuming soup.

### Estimated amount consumed

At the end of each session, subjects estimated the amount they had consumed. They were given a jug containing 2 kg soup and six soup bowls (250 g). Subjects filled the bowls with the amount of soup they thought they had consumed. The estimated amount consumed was calculated by weighing the jug before and after estimation.

### Appetite, hedonic ratings and questionnaires

Subjects rated feelings of hunger, fullness, and thirst on a 9-point scale from “not at all” (0) to “very much” (9). This was rated before and directly after intake, and 1 hour, 2 hours and 3 hours after ad libitum intake.

Before and after intake, subjects were served a small sample of 10 g soup and rated pleasantness and desire-to-eat the soup on a 100 mm visual analogue scale (VAS) that was scaled from “not at all” (0) to “very much” (100).

At the end of the session, subjects indicated reasons for terminating soup consumption. Subjects were asked to what extent they agreed with the propositions: “I terminated consumption because I was full”, “I terminated consumption because the flavor of the soup was not pleasant anymore”, and “I terminated consumption because I did not like the manner of consumption”. The propositions were answered on a 5-point scale from “totally disagree” (1) to “completely agree” (5).

### Standardization of satiety

To standardize the satiety state, subjects always started the lunch session at the same time. They were instructed to consume the same breakfast and to only drink water before lunch started. Moreover, they were asked to refrain from drinking one hour before lunch. After each lunch, subjects answered questions about what they ate for breakfast and if they ate or drank between breakfast and lunch. Subjects were instructed not to eat until three hours after the lunch to rate subjective satiety.

### Statistical analyses

Statistical analyses were performed using SAS version 9.1.4 (SAS Institute Inc., Cary, NC, USA). Data were presented as means ± SDs. *P*-values of <0.05 were considered significant.

Effects of sip size (small-sip vs. large-sip) on sensory characteristics were assessed in a within-subjects ANOVA (PROC GLM, SAS). Effects of sip size (small-sip vs. large-sip vs. free-sip) and distraction on ad libitum intake, estimated amount consumed, appetite ratings, and reasons to terminate consumption, were assessed in a two-way within-subjects ANOVA (PROC GLM, SAS). The differences between the ad libitum intake and the amount consumed were assessed per condition in a within-subjects ANOVA (PROC GLM, SAS). The accuracy of the estimations was assessed by the absolute difference between the ad libitum intake and the estimated amount consumed in percentiles. Effects of distraction on meal duration, sip size, number of sips, and sip frequency were assessed in a within-subjects ANOVA (PROC GLM, SAS). Gender and order of presentation affected most parameters and were added in the ANOVA models. Parameters not normally distributed were log-transformed before assessment. Tukey-Kramer was used for all post hoc comparisons.

## Results

### Sensory characteristics

**Table 1** shows the sensory characteristics of the soup as rated in the small-sip and large-sip conditions. The sip size did not affect sensory characteristics and the pleasantness of the soup. In addition, the “expected satiation” value was not affected by the sip size.

**Table 1:** Sensory characteristics of the soup consumed in the small-sip and large-sip conditions.^1^ ^2^

	Small-sip	Large-sip	F (1, 50)	P
Pleasantness	57±20	60±19	2.5	0.12
Flavor intensity	59±15	59±15	0.1	0.74
Saltiness	52±18	49±19	2.0	0.17
Thickness	34±17	34±18	0.8	0.38
After-taste intensity	52±20	55±16	0.9	0.34
Expected satiation	46±20	43±17	2.5	0.12

1 Values are means ± SDs, n = 54.

2 Scores were rated on a 100 mm VAS after 45 g of soup in the practice session (first session).

### Ad libitum intake of soup

**Figure 2:**
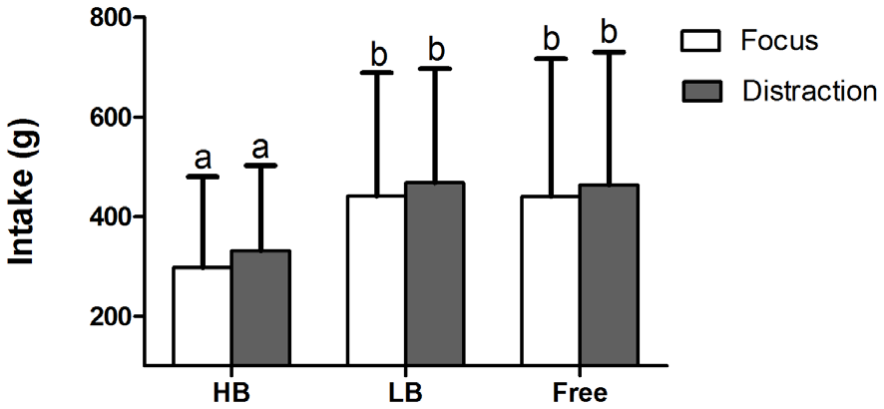
Ad libitum intake in small-sip, large-sip and free-sip conditions (means+SD). Ad libitum intake was higher in the large-sip and free-sip conditions compared to the small-sip condition (P<0.001), and was higher in the distracted state than in the focused state (P = 0.003). Values on bars with different superscript letters are significantly different (P<0.05).

The ad libitum intake in the small-sip condition was ∼30% lower than in the large-sip and free-sip conditions (F(2, 254) = 64, P<0.001), in both the focused and distracted states (Figure 2). The ad libitum intake in the large-sip and free-sip conditions did not differ (P = 0.32). The ad libitum intake was 5 to 11% higher when subjects were distracted than when they were focused (F(1, 254) = 9.0, P = 0.003). There was no interaction between sip size and distraction on ad libitum intake (P = 0.74). In the distracted state, subjects answered 85±12% of the questions correctly (min – max: 50–100%). This outcome was not different between the different sip-size conditions (P = 0.39).

### Estimated amount consumed

**Figure 3:**
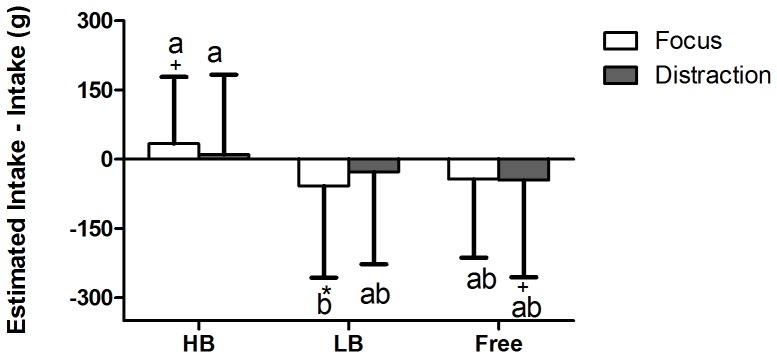
Differences between the estimated amount consumed and the ad libitum intakes (means+SD). The difference (estimated intake minus intake) was affected by sip size (P<0.001), but not by distraction (P = 0.72), and there was no interaction (P = 0.34). * = significant difference between estimated intake and intake (P<0.05., + = trend between estimated intake and intake (P<0.10). Values on bars with different superscript letters are significantly different (P<0.05).

The estimated amount consumed was correlated with the ad libitum intakes (r = 0.66, P<0.001). The direction of the estimations, negative (underestimation) or positive (overestimation), was affected by sip-size (F(2, 254) = 8.3, P<0.001), but not by distraction (P = 0.72) (Figure 3). There was no interaction effect (P = 0.34). Taking into account that distraction did not influence the estimations, subjects significantly underestimated how much they had consumed in both the large-sip condition (both focused and distracted state) (P = 0.030), and the free-sip condition (P = 0.019). Estimations in the small-sip condition did not significantly differ from ad libitum intake (P = 0.16).

The mean values of the estimations in the small-sip condition were 332±190 g in the focused state, which is 11% higher than ad libitum intake (difference between ad libitum intake and estimation: P = 0.09), and 342±175 g in the distracted state, which is 4% higher than ad libitum intake (P = 0.66). The estimations in the large-sip condition were 386±206 g in the focused state, which is 13% lower than ad libitum intake (P = 0.04), and 441±208 g in the distracted state, which is 6% lower than ad libitum intake (P = 0.33). The estimations in the free-sip condition were 397±227 g in the focused state, which is 10% lower than ad libitum intake (P = 0.07) and 419±202 g in the distracted state, which is also 10% lower than ad libitum intake (P = 0.12).

The mean difference in absolute values between the estimated amount consumed minus the ad libitum intake over all conditions was 134±131 g (min-max: 0.1–808 g). Estimation accuracy (i.e., the absolute difference in percentiles between estimated amount consumed minus ad libitum intake) did not differ between the sip-size conditions (P = 0.36) and did not differ between the distracted and focused states (P = 0.88). There was a significant gender effect (P = 0.018); women were 5% more accurate in their estimations than men.

### Distraction, sip size, and estimations in the free-sip condition

In the free-sip condition, subjects determined their sip sizes and sip frequency by themselves. The sip size was not affected by distraction (Table 2). The total number of sips was 11% higher in the distracted state than in the focused state. In the distracted state, the total duration of ad libitum intake was longer, and the eating rate and sip frequency were lower.

**Table 2:** Duration, sip size, number of sips, and sip frequency in the free-sip condition.^1^

	Free-sip		F (1, 53)	P
	Focus	Distraction		
Total duration (min)	6.2±3.7	8.1±4.1	16	<0.001
Eating rate (g/min)	72.0±19.1	60.3±23.4	24	<0.001
Sip size (g)	14.3±5.8	13.5±4.8	2.3	0.13
Total number of sips	32.3±18.6	36.0±18.9	5.7	0.021
Sip frequency (bites/min)	5.6±2.1	4.8±1.9	13	<0.001

1 Values are means ± SDs, n = 53.

In the free condition, subjects determined sip size by themselves, this ranged from 4.0 to 32.1 g. The data set of the free-sip condition (n = 106) has been split up in two groups, a small-sip group (9.9±2.2 g, n = 53) and a large-sip group (17.9±4.4 g, n = 53). The large-sip group showed an underestimation of 87.4±164 g (ad lib intake – estimated intake) (P<0.001), whereas small-sip group did not 0.80±205 g (P = 0.98) (difference between groups: F(1, 104) = 5.8, P = 0.018).

### Appetite and hedonic ratings

**Figure 4:**
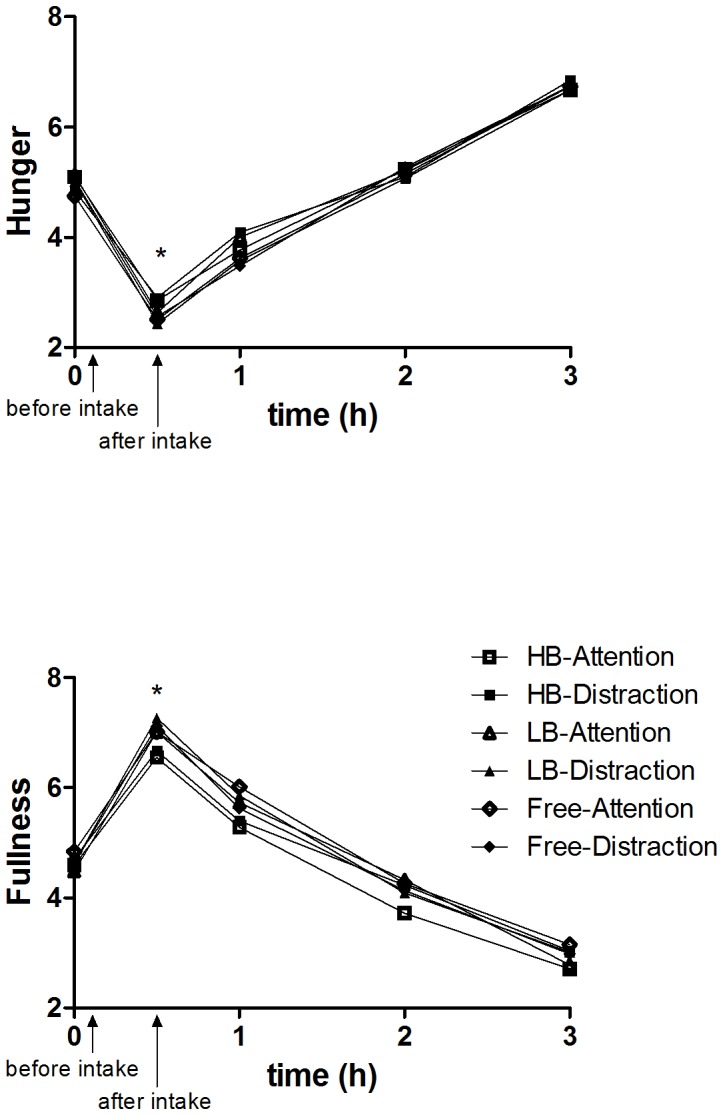
Hunger (A) and fullness (B) ratings over time (9-point scale) (means). *After ad libitum intake (t = 0.5 h), hunger and fullness were affected by sip size (P<0.004), but not by distraction (P>0.31). * The ratings for hunger (A) were higher after the small-sip condition compared to the large-sip condition (P<0.003). The ratings for fullness (B) were lower after the small-sip condition compared to both the large-sip and the free-sip conditions (P<0.009).

Initial ratings of hunger, fullness and prospective consumption did not differ between conditions (all P-values>0.75), indicating that subjects were in the same state of satiety before ad libitum intake in each condition (Figure 4).

After ad libitum intake (t = 30 min), hunger (Figure 4A) was affected by sip size (F(2, 241) = 5.7, P = 0.004), but not by distraction (P = 1.0). Hunger ratings were higher after the small-sip condition, compared to the large-sip condition (P = 0.003) and tended to be higher compared to the free-sip condition (P = 0.06). Hunger was not affected by sip size after 1, 2, and 3 hours (P>0.32). Likewise, ratings for fullness (Figure 4B) were affected by sip size after ad libitum intake (t = 30 min) (F(2, 241) = 11, P<0.001), but not by distraction (P = 0.31). The ratings for fullness were lower after the small-sip compared to both the large-sip and free-sip conditions (P<0.009). Fullness was not significantly affected by sip size after 1 hour (P = 0.07), 2 hours (P = 0.11), and 3 hours (P = 0.70).

Decrease in pleasantness and desire-to-eat the soup after ad libitum intake (data not shown) was not affected by sip size (P>0.33), or distraction (P>0.52).

### Reasons to terminate consumption

“I terminated consumption because I was full” was the most important reason in all conditions to terminate consumption (**Table 3**). All three reasons to terminate consumption were affected by sip size (P<0.012), but not significantly by distraction (P>0.07). The importance of the reason “I terminated consumption because I did not like the manner of consumption” differed between all three sip size conditions (F(2, 249) = 33, P<0.001): small-sip>large-sip>free-sip. The reasons “I was full” and “Flavor was not pleasant anymore” were more important in the large-sip and free-sip conditions compared to the small-sip condition (P<0.036).

**Table 3:** The reasons to terminate soup consumption.^1^ ^2^

	Small-sip		Large-sip		Free-sip		F(2, 249)	P
	Focus	Distraction	Focus	Distraction	Focus	Distraction		
“I was full”^3^	3.7^a^±1.1	4.0^ab^±1.0	4.0^ab^±1.0	4.2^b^±0.9	4.2^b^±1.0	4.2^b^±0.9	7.6	<0.001
“Flavor not pleasant”^3^	2.6±1.1	2.8±1.3	3.0±1.2	2.9±1.2	3.1±1.2	3.0±1.2	4.5	0.012
“Manner of consumption”^3^	3.2^a^±1.3	3.2^a^±1.2	2.7^b^±1.2	2.6^bc^±1.2	2.5^bc^±1.0	2.2^c^±0.9	33	<0.001

1 Values are means ± SDs. Values in rows with different superscript letters are significantly different (P<0.05).

2 The propositions were answered on a 5-point scale from “totally disagree” (1) to “completely agree” (5).

3 Significant main effects of sip size P<0.012.

To assess whether ratings of “manner of consumption” would overrule effects of sip size on ad libitum intake, we added these ratings as covariate in the statistical model that tested effects on ad libitum intake. Ratings of “manner of consumption” affected ad libitum intake (F(1, 248) = 9.5, P = 0.002). However, there was still a large effect of sip size on ad libitum intake: (F(2, 248) = 41, P<0.001) (and also an effect of distraction on ad libitum intake: (F(1, 248) = 7.0, P = 0.008)). This means that with correction of ratings of the manner of consumption, intake in the small-sip condition remained significantly lower compared to the large- and free-sip conditions.

## Discussion

### Effect of sip size on food intake and on the estimated amount consumed

We hypothesized that ad libitum intake would be higher when subjects consume large sips and that they would underestimate how much they had consumed. The results showed, indeed, that ad libitum intake was higher when consuming large sips, in agreement with previous studies [4], [6], [7], [13]–[18]. Consuming large sips led to underestimation, whereas small sips, led numerically, but not significantly, to overestimation of the amount consumed. This indicates that sip size affects the perceived food intake. Larger sips are by definition associated with fewer sips per gram food. The fact that fewer sips are consumed, may explain the underestimation of food intake in the large-sip condition. This underestimation during consumption may delay satiation, because food intake is influenced by believes about the amount of food intake [25].

Interestingly, when subjects determined their sip size and frequency by themselves (free-sip condition), ad libitum intake was similar to the large-sip condition. Subjects also underestimated how much they had consumed in the free-sip condition. Moreover, subjects consumed soup in the free-sip condition with almost similar sip size than the large-sip condition (∼14 g and 15 g, respectively). Dividing the data of the free-sip condition into two groups (small-sip and large-sip), showed that taking large sips lead to an underestimation whereas smaller sips did not. This indicates that underestimation of consumption also occurs when people take relatively large bites by themselves.

The mean sip size in the free-sip condition of ∼14 g is larger than the sips that are taken when the soup is consumed with spoons: 7–9 g [44]. The sip size was probably influenced by the manner of consumption, which was through a tube. It has been shown that consuming with a straw instead of a spoon increased eating rate, possibly through relatively large sips facilitated by straws [45]. The tube may therefore facilitate large sips compared to spoons.

The reason “I terminated consumption because I did not like the manner of consumption” was more important in the small-sip condition compared to the large-sip and free-sip conditions. It probably contributes to the 30% lower intake in the small-sip condition. The lower intake in the small-sip condition may also explain why subjects felt less full directly after consumption. However, when the statistical model on ad libitum intake was corrected for “manner of consumption”, there is still a strong significant effect of sip size on ad libitum intake.

Subjects felt less full after consumption in the small-sip condition compared to the large-sip and free-sip conditions. However, these differences in hunger and fullness ratings diminished at one to three hours after consumption (Figure 4). No differences in hunger after three hours may indicate that the reduced food intake in the small-sip condition will not be compensated. Two studies [16], [46], that used an oral device to decrease bite sizes, have shown that the device led to a reduction in meal size without changes on rated satiety between meals. Small bites or sips may therefore lead to a reduction in food intake on longer term.

Sip size did not influence sensory characteristics of the soup (Table 1). In addition, the initial pleasantness and the decrease in pleasantness after ad libitum intake were not affected. Therefore, the effect of sip size on ad libitum intake was not mediated via differences in flavor perception or pleasantness of the food.

Larger sips are associated with fewer sips per gram food. Fewer sips means less effort, which facilitates food intake. Effort is related to the ease with which a food can be consumed and has a strong influence on the amount of food intake [47], [48]. For example, a longer distance to a snack product increase the effort to obtain the snack and this was shown to reduce energy intake [49]. We observed that subjects took relatively large sips when they chose the size themselves. Peoples' natural behavior act through the “Law of least effort” [50]. Consuming with large sips or bites means that people chose the path of least effort or resistance.

Effect of sip size on food intake may not only externally regulated but also internally. In a previous study, we showed that large sips decrease the relative oral sensory exposure to the taste of the food (i.e., exposure to taste per gram food) [10]. Oral sensory exposure has been shown to play an important role in the development satiation [3], [4], [51]–[54]. In addition, also a higher number of swallows that is associated with smaller sips or bites, may play a role in the onset of signals of satiation [55].

### Effects of distraction on food intake, estimated amount consumed, and sip size

Distraction led to greater intake (5–11%), in agreement with a number of studies [34]–[39]. Other studies have found an increase in energy intake of ∼14% when watching TV [34], [37]. This is somewhat greater than the effect found in the present study. Others suggested that the increased food intake in distracted states is explained by impaired ability to visually monitor the amount consumed [25], [37], [40]. This study differed from others because subjects were not able to visually monitor the amount consumed. Therefore, it is possible that impaired visual cues play a role, but there must be other mechanisms that explain increased food intake during distraction.

Distraction led to lower sip frequency and longer meal duration in the free-sip condition. In addition, distraction was associated with higher number of total sips, whereas sip size was not affected. Sip size may be an individual behavioral characteristic that is not influenced by distraction. This is in agreement with the finding that bite size is constant within individuals for specific types of food [20], [21], [56], which is probably also the case for sip size.

Another study [39] also showed prolonged meal duration and increased food intake when people were distracted by listening to music. The present study also showed that the distracted state slowed down eating rate but prolonged meal duration that resulted in higher food intake. Longer meal duration, thus more opportunity to eat, may explain increased food intake in distracted states. It is also possible that the sensory exposure per gram food is less in distracted states. Watching the film distracted attention away from oral food processing. Oral sensory exposure to food is important for termination of food consumption (e.g., [3], [53]).

To ensure subjects were distracted, they watched an animation film during consumption and were instructed to answer questions afterwards. The distraction was successful because these questions were well-answered. The minimum score was 50% correct (out of 8 to 11 questions). These questions could not be answered if no attention was paid to the film.

We hypothesized that the effect of sip size on food intake is diminished in a distracted state. Distraction did not influence the effect size of sip size on food intake; there was no interaction effect. This means that the effect of reducing intake by consuming small sips is not overruled by increasing the number of sips in a distracted state. Therefore, smaller sip sizes are effective in reducing food intake even when people are distracted.

We hypothesized that subjects would underestimate how much they had consumed when they were distracted. The results showed that both the direction and the accuracy of the estimated amount consumed were not affected by distraction. This contradicts a recent study that showed distraction resulted in impaired memory for the consumed foods [35]. In that study, subjects had to recall the different lunch items they ate after 30 minutes, which is different from estimating the amount consumed directly after intake. The results of the present study suggest that in a distracted state without visual cues, people somehow know how much they approximately consumed. Probably, their attention to the film did not completely diminish attention towards food consumption.

### Conclusion

Consumption with large sips, thus relatively fewer sips per gram food, led to much higher food intake and led to an underestimation of the amount consumed. When subjects were able to determine sip sizes by themselves, they took relatively large sips and also underestimated the amount consumed. Underestimating the amount consumed is a possible risk factor for overconsumption. Distraction led to a general increase in food intake, independent of sip size. In addition, subjects did not adjust their sip sizes when they were distracted. This implies that small sips or bites may successfully reduce food intake, even in a distracted state. Designing foods by food industry that involves consumption of small bites/sips may prevent overconsumption and decrease the prevalence of obesity.
